# Etiology of UTI – pathogens involved and their sensitivity to antibiotics

**DOI:** 10.1186/1471-2334-14-S7-P33

**Published:** 2014-10-15

**Authors:** Sorina Carp, Anca Dumitrescu, Sorin Rugină, Elena Dumea, Maria Margareta Ilie, Stela Halichidis, Simona Claudia Cambrea

**Affiliations:** 1 Clinical Infectious Diseases Hospital of Constanța, Romania; 2 Faculty of Medicine, “Ovidius” University, Constanța, Romania

## Background

The study aims to evaluate the etiologic spectrum of urinary tract infections (UTI) and antibiotic sensitivity of isolates.

## Methods

At the Infectious Disease Hospital in Constanța, between January 2014 and June 2014, urine cultures were performed in patients presenting urinary tract infections. Urine was seeded by loops calibrated technique on Columbia agar with 5% sheep blood and Drigalski agar. To identify the germs we used latex agglutination kits for Gram-positive germs and API galleries (BioMerieux) for Gram-negative bacilli. Antibiotics susceptibility testing was performed with the help of Kirby-Bauer disc diffusion method.

## Results

Out of the total 805 urine samples performed, 160 turned out positive, isolating in 94 cases (58.7%) *Escherichia coli*, 25 cases (15.6%) *Klebsiella* spp., 12 cases (7%) *Proteus* spp., 15 cases (9.3%) *Enterococcus* spp., 7 cases (4.3%) *Staphylococcus* spp. and others – 7 cases (4.3%). *E. coli* was the most common etiological agent of UTI in women (64% of positive cases), men (42.3% of positive cases) and children (47% of positive cases).

Most of the isolated germs were highly susceptible to imipenem (94.4%) and fosfomycin (82.28%). Also, a good sensitivity to ceftriaxone (69.2%), quinolones (71.4%) and gentamicin (68.68%) was recorded. In addition, 56.6% of germs were sensitive to nalidixic acid and 56.6% to biseptol. We also noticed low sensitivity of germs to amoxicillin/clavulanic acid – 32.7%.

## Conclusion

*Escherichia coli* was found in the highest percentage in urinary isolates, predominantly in women. There is an increased sensitivity of strains involved in urinary tract infections to fosfomycin – an affordable antibiotic that can be used in the treatment of these cases.

